# Parkinson's Disease Gravity Index: A Method by means of Optimal Scaling

**DOI:** 10.1155/2020/8871870

**Published:** 2020-12-15

**Authors:** Marcos Serrano-Dueñas, Luis Masabanda, Maria-Rosario Luquin

**Affiliations:** ^1^Facultad de Medicina, Pontificia Universidad Católica del Ecuador, Quito, Ecuador; ^2^Servicio de Neurología, Hospital Carlos Andrade Marín, Quito, Ecuador; ^3^Departamento de Neurología, Clínica Universidad de Navarra, Pamplona, Spain

## Abstract

**Objective:**

This study has been designed with the aim of using optimal scaling to perform the allocation of scores and to be able to construct an indicator of the Parkinson's Disease Gravity Index. Scores were assigned to interrelated dimensions that share information about the patient's situation, to have an objective, holistic tool which integrates scores so that doctors can have a comprehensive idea of the patient's situation. *Patients and Methods.* 120 consecutive patients with Parkinson's diagnosis were chosen according to the United Kingdom Parkinson's Disease Society Brain Bank criteria. Subsequently, all the chosen dimensions were transformed into interval variables for which the formula proposed by Sturges was used. Once the dimensions were transformed into interval variables, optimal scaling was carried out. Subsequently, the following attributes were analyzed: quality and acceptability of the data; reliability: internal consistency, reliability index, Cronbach's alpha, and standard error of measurement; finally, validity: convergent validity and validity for known groups.

**Results:**

There were no missing data. An appropriate Cronbach's alpha value of 0.71 was gathered, and all items were found to be pertinent to the scale. The item homogeneity index was 0.36. Precision evaluated with the standard error of measurement was 7.8. The Parkinson's Disease Gravity Index discriminant validity (validity for known groups), assessed among the different stages of Hoehn and Yahr scale by the Kruskal–Wallis test, showed major significance (*X*^2^ = 32.7, *p* ≤ 0.001).

**Conclusions:**

The Parkinson's Disease Gravity Index has shown adequate metric properties.

## 1. Introduction

Parkinson's disease (PD) is a heterogeneous neurodegenerative process, which, by 2016, was estimated to affect some 6.1 million people [1].

The onset of neurodegeneration of Parkinson's disease is likely to occur several decades before the onset of motor symptoms. Possible risk factors include genetic predisposition and environmental factors such as exposure to toxins. Parkinson's disease is characterized by a selective loss of dopaminergic neurons in the substantia nigra *pars* compacta; subsequently, there is a widespread involvement of other circuits of the central and peripheral nervous system. Changes associated with genomic, epigenetic, and environmental factors lead to structural alterations and protein deposits, especially alpha-synuclein, due to dysfunction in the ubiquitin-proteasome system, alteration of mitochondrial function, and oxidative stress [2].

PD is more than just a movement disorder. Other PD subtypes have been proposed, such as autonomic dysfunction, cognitive deterioration, and REM sleep disorder. However, there is still a clear emphasis on the motor symptoms associated with PD [3]. There are different degrees of nonmotor symptoms of the disease (NMS) such as autonomic dysfunction, sensory disorders, integumentary system disorders, neurobehavioral disorders, sleep disorders, visual impairment, and other conditions [4].

We should point out that there is no clear correlation between motor involvement and nonmotor disorders. In a study of 935 patients, Ray Chaudhuri et al. [5] concluded that although the NMS (Nonmotor Symptoms Scale (NMSS)) [6] increases with the severity of PD, the correlation between motor disorder and the NMS was moderate (Spearman's *ρ* (rhoS = 0.43)). This shows that the association between motor disorder and NMS varies at all stages of the disease, except for in the most advanced cases. Most importantly, there is a clear incongruity between motor and nonmotor abnormalities as patients in the milder stages of motor disorder may have considerable nonmotor symptoms. For example, in the study by Ray Chaudhuri et al. [5], more than a third of the patients (34.5%) were in stages 1 and 2 of the Hoehn and Yahr (H&Y) scale [7] and presented severe or very severe nonmotor symptoms.

The International Parkinson and Movement Disorders Society offers MDS-owned rating scales, translated scales, and a listing of other recommended rating scales to carry out the evaluation of all the previously mentioned conditions. Using these scales, by the end of the evaluation of patients, a series of scores of different amounts are generated.

These scores should be considered by the doctor. However, the doctor, in a personal and subjective manner, should assign their own scores based on their experience, with the goal of reaching a total score which is a comprehensive view of the situation of the patient, and create an appropriate treatment plan. There is a published proposal to perform the Parkinson's Disease Composite Scale (PDCS) that uses heterogeneous values to assess and categorize the severity of symptoms [8]. The authors used the disease staging according to the Hoehn and Yahr (H&Y) scale and various scores in sections of the Clinical Impression of Severity Index for Parkinson's Disease (CISI-PD): the Movement Disorder Society-Unified Parkinson's Disease Rating Scale (MDS-UPDRS), the Nonmotor Symptoms Scale (NMSS), or the Nonmotor Symptoms Questionnaire (NMSQuest).

However, a core issue remains unanswered: how should doctors assess the importance of symptoms? There are such diverse symptoms which belong to quite different fields, for example, the anxiety section of the HADS (a construct, evaluated by the patient himself) which has a maximum score of 21 points and the motor SCOPA (a scale based on the examiner's scores) which has a maximum score of 75. If the patient scores 7 points of anxiety, is it more (or less) important than 25 points of motor SCOPA? Similar questions can be asked of the other scales.

On the other hand, we must assume that the scores of the scales are actually discrete scores, that is, that in a strict sense, they do not admit fragmentation and only positive integers belonging to the set of real numbers (natural numbers) are possible, and therefore, they could not be divided.

Given the dispersion of the scores, we should treat them as interval variables (class intervals) (by classifying, ordering, establishing a distance between the values, and having a unit of measurement). In this type of measurement, the numbers assigned to the objects have all the characteristics of ordinal measurements, and the differences between the measurements represent equivalent intervals. Once these intervals have been created, we can consider them as ordinal categorical variables (classification and sorting), which must also be exhaustive, exclusive, and referenced to a single classifying principle [9].

Due to this, it is necessary to have a mathematical algorithm that allows us to take all the scores and transform them so that, in the end, we have comparable scores.

Fisher called this methodology “the appropriate scoring technique” [10]. Later, Young made the optimal scaling (OS) proposal [11, 12] as the appropriate mathematical method for transforming scores.

Briefly, if we have a matrix **X** that contains **n** individuals and **m** variables, that is to say, a matrix **n × m**, OS is a multivariate analysis technique, which analyses the relationship of the **n** random variables and allows for reducing the size of the data matrix with a minimum loss of information. Many of those **m** variables are categorical in nature (nominal or ordinal). The idea is to assign quantitative values to these categorical variables using nonlinear methods since not all **m** variables are linearly correlated with each other.

Since, in practice, multivariate systems never have maximum homogeneity (variables can share information, but not totally), the reduction of dimensions always involves loss of information, which can be measured through a loss function. The functions most used for this purpose are made by averaging the sums of the squares of the differences between the score vector of the individuals and each of the variables.

This is equivalent to gaining the vector **Y** as the summary of the variables previously scaled or linearly transformed, so that each is weighted according to the amount of information shared with the other variables. The transformations that guarantee that the summary vector (score vector of the individuals) collects as much information as possible are usually gained based on the breakdown of values and eigenvectors of the matrix [13–15].

For this reason, this study has been designed with the aim of using OS to perform the allocation of scores and to be able to construct an indicator of the Parkinson's Disease Gravity Index (PDGI). The study's objective is to assign scores to interrelated dimensions that share information about the patient's situation, in order to have an objective, holistic tool which integrates scores so that doctors can have a comprehensive idea of the patient's situation.

## 2. Patients and Methods

To calculate the sample size, parameters suggested by Beavers et al. [16] were applied, and the UKPDSBB clinical diagnosis criteria [17] were used to select the one hundred and twenty patients with PD who participated in the study. All patients were attended at the Ambulatory Setting of the Unit of Abnormal Movements of the Neurology Service of the Carlos Andrade Marín Hospital, Quito, Ecuador.

All patients gave their informed consent to participate in the study, which was approved by the Teaching and Research Department of the Carlos Andrade Marín Hospital and by the Bioethics Committee of the University of Navarra (Spain).

The exclusion criteria involved the presence of any neurological disorder that caused disability: hemiplegia, blindness, deafness, or the presence of a serious acute illness.

*Patient Evaluations*. All patients were evaluated during the “ON” period.

The following dimensions were included in the evaluation: (1) age dimension (AD) in years, (2) motor dimension (MD) evaluated with SPES-SCOPA [18], (3) depression dimension (DD) evaluated with HADS [19], (4) anxiety dimension (AxD) with the same, (5) cognitive dimension (CD) evaluated with PD-CRS [20], (6) apathy dimension (ApD) measured with AS [21], (7) fatigue dimension (FD) with D-FIS [22], (8) nonmotor dimension (NMD) with the NMSS [23] (except for the following domains: sleep, fatigue, mood, apathy, perceptual problems, hallucinations, attention problems, and memory), (9) psychosis dimension (PsD) measured with SCOPA-PC [24], and (10) sleep dimension (SD) evaluated with SCOPA-SLEEP [25].

Demographic data of interest were collected. Besides the rating scales indicated, the stages of the disease were evaluated using the H&Y scale [26]. The Schwab and England (S&E) scale was used to study daily life activities [27]. The PIMS was used to assess the quality of life [28], and finally, the CISI-PD [29] was used to make clinical information of the severity of the disease.

### 2.1. Method of Analysis

The PDGI contains the 10 dimensions indicated above: (1) age dimension (AD), (2) motor dimension (MD), (3) depression dimension (DD), (4) anxiety dimension (AxD), (5) cognitive dimension (CD), (6) apathy dimension (ApD), (7) fatigue dimension (FD), (8) nonmotor dimension (NMD), (9) psychosis dimension (PsD), and (10) sleep dimension (SD).

Descriptive statistics of the central tendency and dispersion were gathered. Subsequently, all the chosen dimensions were transformed into interval variables, for which the formula proposed by Sturges [30] was used: *K* = *R*/(1 + 3,322 ∗ log *N*), where *K* is the number of intervals, *R* is the range, and log *N* is the natural logarithm (base 10) of *N*, which is the number of individuals. Then, we calculated *W* = *K*/*R*, where *W* is the width/width of each interval.

Once the dimensions were transformed into interval variables, OS was carried out. For this, the statistical package SPSS.v.17 was used.

After retrieving the initial values of the intervals, OS was carried out, which assigns a value to each interval. We collected the minimum (the value of which is absolute) and added to each of the previous values, and we were left with what we call the “corrected value of each interval.”

From each dimension, we took the “maximum corrected value of each interval” and added them. In our case, there were 10 dimensions, the sum of which reached 36.44. Since we know that the maximum theoretical value is 100, we divided it by that sum (100/36.44) = 2.744, which is the “index factor.”

We multiplied the “index factor” by each of the “corrected values of each interval” and thus found the new “corrected values multiplied by the factor.” For confirmation, if we add up the “maximum corrected values multiplied by the factor,” we will get 100 (model) (Table 1).

For example, for a patient who has an original score with the SPES-SCOPA in the interval of 24–30 points, the corrected value is 1.18. When multiplied by the factor of 2,744, the final value is 3.24 points, which is the number used in the PDSI (Table 2).

#### 2.1.1. Data Quality and Acceptability

(i) Lost data must not exceed 5%; (ii) the difference between the average and median should not exceed 10% of the highest possible score; and (iii) the floor and ceiling effects must not exceed 15% [31]. The skewness and kurtosis coefficients must lie within the interval of −1, 1 [32].

#### 2.1.2. Reliability

(i) Internal consistency: the homogeneity index of the items must be ≥0.330; and (ii) the reliability index, Cronbach's alpha (C'*ɑ*) value, must be greater than 0.70 [33], and the standard error of measurement (SEM) was attained; the SEM must be equal to the standard deviation, multiplying by the square root of 1 minus C'*ɑ* = (SD ∗ √1 − reliability coefficient) [34], where SD indicates standard deviation. We compared the SEM with half the amount of the standard deviation and gained a lower value, thus yielding a precision of ≥75% [35].

#### 2.1.3. Validity

(i) Convergent validity: for this, Spearman's correlation coefficient (rhoS) and the values suggested by Akoglu [36] were used (0 = no correlation; 0.1–0.3 = weak correlation; 0.4–0.6 = moderate correlation; 0.7–0.9 = strong correlation; 1 = perfect correlation) and (ii) validity for known groups: for this, we used the H&Y stages as a segmentation variable, and a value ≤0.05 was observed as significant.

## 3. Results

There were 120 patients with a mean age of 68.5 years, 9 years of illness, with 683.5 ± 225.5 mg of levodopa/day (or equivalent dose of levodopa); 60.8% of patients were males. The same number of patients were retired. Seventy-four (61.7%) were in stage III of the H&Y classification (Table 3). The mean ± (sd) scores of the variables that make up the Parkinson's Disease Gravity Index are shown in Table 4.

All the details of the OS results are presented in Table 2. With the OS method, the highest value reached was for the PsD which is 12.84, and the lowest ApD is 7.52 (Table 2).

Once the transformation of the scores of the variables studied was carried out using the OS, the PDSI analytical study was carried out.

### 3.1. Data Quality and Acceptability

There were no missing data; all the information was analyzed. When analysing the items' metric characteristics, we found that PsD has a big floor effect (61.6%) and a kurtosis of 3.3 (Table 5).

### 3.2. Reliability

An appropriate C'*ɑ* value of 0.71 was gathered, and all items were found to be pertinent to the scale. The item homogeneity index was 0.36 (standard value: >0.3). Precision evaluated with the SEM (SEM = SD × √1 − reliability coefficient = 14.5 × √1 − 0.71 = 7.8, a little higher than 7.25 (14.5 × 0.5)) was acceptable.

### 3.3. Validity

Convergent validity, assessed by Spearman's rho (rhoS) correlation between the PDSteI and the S&E, was moderately correlated (rhoS: −0.69), with PIMS (rhoS: 0.62) and CISI-PD total (rhoS: 0.58). (Table 6).

**Figure 1:**
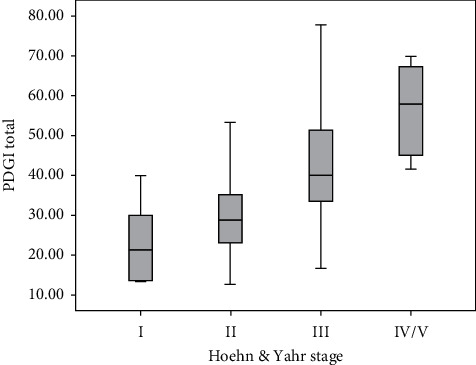
Boxplot of the PDGI total for the H&Y staging.

The scale's discriminant validity (validity for known groups), assessed among the different stages of the H&Y scale by the Kruskal–Wallis test, showed major significance (*X*^2^ = 32.7, *p* ≤ 0.001) (Figure 1).

## 4. Discussion

In the study, the population was characterized by having a wide representation in stages II and III (34 and 74 patients, respectively) and 9.5 years of disease on average. This is a very common sample in outpatient studies [37].

Regarding the acceptability of the PDGI items, we can point out that, in five items, the floor effect was observed: ApD (27.5), FD (21.6), NmD (21.6), PsD (61.6), and SD (15.8). Similar to what was found by Martinez–Martin et al. [37] with their PDCS, the problem of PsD in outpatients is that its prevalence is generally low. For example, Visser et al. [24], using the same evaluation tool, found that only 21 (3%) of the patients studied had symptoms of this dimension. In the study by Martinez–Martin et al. [37], where the PsD was referred to as hallucinations, they found a floor effect greater than 70%.

None of the patients presented the ceiling effect. As for the asymmetry, only the PsD was outside the norm value (1.6), which evidences its high floor effect/consequent data of its high floor effect. Regarding kurtosis, the CD, AD, and NmD dimensions had minimum values outside the allowed range (−1.2, −1.34, and −1.3, respectively). The PsD presented a value of 3.3 (Table 5).

The corrected item-total correlation ranged from 0.16 for the AD to 0.72 for the FD. Regarding the C'*ɑ*, if the item is deleted, then the item that contributes the most to the value of the alpha is the FD; without that item, the alpha fell to 0.61. The item that contributes the least is the CD; when it was removed, the alpha rose to 0.82. In these circumstances, the range between the alpha values was 0.2.

To perform the convergent validity analysis, we found that the AD had generally weak correlations with the variables S&E, PIMS, and CISI-PD total (values of −0.25, 0.10, and 0.24, respectively). The PsD showed similar behaviour, with *d* −0.31, 0.2, and 0.2 values, with those same variables (Table 6).

Meanwhile, the MD reached moderate and strong correlation values: −0.76 against the S&E, 0.56 with PIMS, and 0.82 with CISI-PD total. The rest of the dimensions had moderate values. The total PDGI was correlated with moderate values: −0.69 with S&E, 0.62 with PIMS, and 0.58 with CISI-PD total. In the work of Martinez–Martin et al., the PDCS reached values of 0.76 and 0.89, respectively, compared to PDQ-39, which measures the quality of life and before the CISI-PD total [37].

Compared to other demographic variables such as years of disease and levodopa dose, the PDGI total had moderate correlation values (0.37 and 0.46, respectively). These correlations are like those gained with the PDCS [37] (Table 6).

This PDGI is not considered as a tool for use in daily practice; its function is to be able to carry out a global and therefore extensive evaluation of the situation of a patient.

Overall, our PDGI has adequate metric properties; acceptability, internal consistency, and convergent validity are adequate. This proposal is more in line with a holistic, inclusive evaluation, it does not assume a greater preponderance of motor symptoms, and it includes the different dimensions that affect people with PD. Likewise, it does not allow subjective appreciation to guide giving more or less weight to any of the dimensions, but rather, an objective mathematical algorithm assigns the scores.

## 5. Conclusions

As noted at the beginning, this method allows each dimension to have a weight according to the amount of information it shares with the other variables and dimensions. This is the method's advantage over existing scales, which do not give values of different scales gathered under a certain category such as mild or severe. The PDGI groups the scores that share information about the subject. Therefore, each score offers part of the clinical situation of a patient, and this allows for a detailed summation of the patient's state.

## Figures and Tables

**Table 1:** OS model.

MD	“Corrected value of each interval” by the OS	Minimum absolute value	“Corrected value of each interval” by the OS	Maximum	Corrected value multiplied by the factor	Maximum
[3–16]	−1.48	1.48	0		0	
[17–23]	−1.01		0.47		1.29	
[24–30]	−0.3		1.18		3.24	
[31–44]	0.9		2.38		6.53	
[45–58]	1.85		3.33	3.33	9.14	9.14

**Table 2:** Transformation with the OS.

			“Corrected value of each interval” by the OS	Minimum absolute value	“Corrected value of each interval” by the OS	Maximum	Corrected value multiplied by the factor	Maximum
AD	1	36–49	−1.85	1.85	0		0	
	2	50–63	−0.79		1.06		2.91	
	3	64–70	−0.6		1.25		3.43	
	4	71–77	0.39		2.24		6.15	
	5	78–84	1.54		3.39		9.3	
	6	85–92	1.68		3.53	3.53	9.69	9.69

MD	1	3–16	−1.48	1.48	0		0	
	2	17–23	−1.01		0.47		1.29	
	3	24–30	−0.3		1.18		3.24	
	4	31–44	0.9		2.38		6.53	
	5	45–58	1.85		3.33	3.33	9.14	9.14

DD	1	0–1.5	−1.58	1.58	0		0	
	2	1.51–3.01	−1.11		0.47		1.29	
	3	3.02–4.52	−0.75		0.83		2.28	
	4	4.53–7.54	0.2		1.78		4.88	
	5	7.55–10.56	1.48		3.06		8.4	
	6	10.57–12.07	2.25		3.83	3.83	10.51	10.51

AxD	1	0–1.75	−1.23	1.23	0		0	
	2	1.76–3.51	−0.89		0.34		0.93	
	3	3.52–7.03	−0.53		0.7		1.92	
	4	7.04–8.79	0.47		1.7		4.67	
	5	8.80–10.55	0.74		1.97		5.41	
	6	10.56–12.31	1.72		2.95		8.1	
	7	12.32–14.07	2.29		3.52	3.52	9.66	9.66

CD	1	16–27.125	−1.71	1.71	0		0	
	2	27.126–49.377	−1		0.71		1.95	
	3	49.378–60.503	−0.12		1.59		4.36	
	4	60.504–71.629	0.66		2.37		6.5	
	5	71.63–82.755	1.25		2.96		8.12	
	6	82.756–93.881	1.39		3.1		8.51	
	7	93.882–105.007	1.65		3.36	3.36	9.22	9.22

ApD	1	0–4.25	−1.21	1.21	0		0	
	2	4.26–8.51	−0.81		0.4		1.1	
	3	8.52–12.77	−0.31		0.9		2.47	
	4	12.78–17.03	0.5		1.71		4.69	
	5	17.04–21.29	0.56		1.77		4.86	
	6	21.3–25.55	1.51		2.72		7.46	
	7	25.56–34.07	1.53		2.74	2.74	7.52	7.52

FD	1	0–3.625	−1.33	1.33	0		0	
	2	3.626–7.251	−0.8		0.53		1.45	
	3	7.252–10.877	−0.03		1.3		3.57	
	4	10.878–14.503	0.71		2.04		5.6	
	5	14.504–18.129	0.88		2.21		6.06	
	6	18.13–21.755	1.32		2.65		7.27	
	7	21.756–25.381	1.97		3.3		9.06	
	8	25.382–29.007	2.39		3.72	3.72	10.21	10.21

NMD	1	2–16.25	−1.29	1.29	0		0	
	2	16.26–30.51	−0.53		0.76		2.09	
	3	30.52–44.77	0.61		1.9		5.21	
	4	44.78–101.81	1.47		2.76		7.57	
	5	101.82–116.07	1.87		3.16	3.16	8.67	8.67

PsD	1	0–1	−0.68	0.68	0		0	
	2	1.01–2.01	0.39		1.07		2.94	
	3	2.02–4.03	1.1		1.78		4.88	
	4	4.04–5.04	1.84		2.52		6.92	
	5	5.05–8.07	3.89		4.57	4.57	12.54	12.54

SD	1	0–2.5	−1.52	1.52	0		0	
	2	2.51–5.01	−0.81		0.71		1.95	
	3	5.02–7.52	0		1.52		4.17	
	4	7.53–12.54	0.62		2.14		5.87	
	5	12.55–17.56	1.2		2.72		7.46	
	6	17.57–20.07	3.16		4.68	4.68	12.84	12.84

Index factor creation (100/36.44 = 2.744)						36.44		100

AD: age dimension; MD: motor dimension; DD: depression dimension; AxD: anxiety dimension; CD: cognitive dimension; ApD: apathy dimension; FD: fatigue dimension; NMD: nonmotor dimension; PsD: psychosis dimension PsD; SD: sleep dimension.

**Table 3:** Descriptive statistics of the sample (*N* = 120).

*Sex*	Male 73 (60.8%)				

Marital status	Married 86 (71.7%)				
	Single 10 (8.3%)				
	Others 24 (20%)				

Employment status	Retired 73 (60.8%)				
	Full-time worker 15 (12.5%)				
	Housewife or househusband 12 (10%)				
	Others 20 (16.7%)				

	Median	Mean ± sd	IQR	*S*	*K*
Years of education	7	9.6 ± 5.2	8	0.5	−0.9
Duration of disease (years)	8	9 ± 5.6	7	1.4	3.3
Duration of therapy with L-dopa (years)	6	7.5 ± 5.3	6.3	1.3	2.7
Dose of L-dopa (mg/day)	750	683.5 ± 225.5	250	−0.1	0.5
PIMS	21	19.9 ± 7	10	−0.5	−0.1
CISI total	10	10.1 ± 4.1	6	0.3	−0.3

IQR = interquartile range; *S *= skewness; *C *= kurtosis; PIMS: Parkinson's Impact Scale; CISI: Clinical Impression of Severity Index for Parkinson's Disease.

**Table 4:** Mean ± sd scores of the variables that make up the Parkinson's Disease Severity Index.

Age dimension	68.6±11.0
Motor dimension	28.1±10.3
Depression dimension	5.4±2.7
Anxiety dimension	6.4±3.6
Cognitive dimension	63.3±18.9
Apathy dimension	12.5±8.9
Fatigue dimension	9.6±6.6
Nonmotor dimension	31.6±18.7
Psychosis dimension	1.5±1.6
Sleep dimension	7.6±4.6

**Table 5:** Acceptability data of the variables of the Parkinson's Disease Gravity Index.

	Min	Max	Median	Mean	SD	Floor	Ceiling	Skewness	Kurtosis
AD	0	9.69	3.49	5.09	2.75	6.66	4.16	0.24	−0.8
MD	0	9.14	3.24	3.8	2.76	10	8.33	0.42	−0.98
DD	0	10.51	4.88	4.24	2.85	6.66	5	0.51	−0.53
AxD	0	9.66	1.92	3.38	2.76	9.16	4.16	0.81	−0.5
CD	0	9.22	6.5	5.69	2.69	2.5	3.33	−0.47	−1.22
ApD	0	7.52	2.47	3.32	2.76	27.5	7.5	0.18	−1.34
FD	0	10.21	3.57	3.65	2.75	20.83	2.5	0.22	−0.79
NMD	0	8.67	2.09	3.53	2.72	21.66	0.83	0.2	−1.3
PsD	0	12.54	0	1.87	2.78	61.66	2.5	1.69	3.39
SD	0	12.84	4.17	4.17	2.76	15.83	1.66	0.16	−0.04
Total	12.65	77.65	36.92	38.71	14.5	0.83	1.66	0.38	−0.35

AD: age dimension; MD: motor dimension; DD: depression dimension; AxD: anxiety dimension; CD: cognitive dimension; ApD: apathy dimension; FD: fatigue dimension; NMD: nonmotor dimension; PsD: psychosis dimension; SD: sleep dimension.

**Table 6:** Correlation between the Parkinson's Disease Gravity Index and other measures.

	Years of disease	Years with L-dopa	L-dopa doses	S&E	PIMS	CISI1	CISI2	CISI3	CISI4	CISI total
AD	0.15	0.18	0.41	−0.25	0.1	0.23	0.26	−0.06	0.44	0.24
MD	0.5	0.55	0.52	−0.76	0.56	0.76	0.72	0.67	0.52	0.82
DD	−0.01	0.07	0.17	−0.46	0.5	0.34	0.42	0.21	0.33	0.38
AxD	−0.02	0.09	0.07	−0.45	0.5	0.36	0.42	0.22	0.22	0.35
CD	−0.23	−0.28	−0.43	0.57	−0.41	−0.45	−0.5	−0.26	−0.81	−0.6
ApD	0.13	0.22	0.35	−0.59	0.41	0.38	0.49	0.18	0.66	0.5
FD	0.1	0.2	0.27	−0.58	0.46	0.43	0.48	0.19	0.59	0.5
NMD	0.17	0.26	0.34	−0.42	0.46	0.3	0.36	0.19	0.26	0.32
PsD	−0.07	0.03	0.12	−0.31	0.2	0.2	0.27	0.08	0.22	0.2
SD	0.29	0.29	0.37	−0.33	0.33	0.33	0.34	0.13	0.37	0.35
Total	0.19	0.31	0.42	−0.69	0.62	0.53	0.62	0.27	0.53	0.58

AD: age dimension; MD: motor dimension; DD: depression dimension; AxD: anxiety dimension; CD: cognitive dimension; ApD: apathy dimension; FD: fatigue dimension; NMD: nonmotor dimension; PsD: psychosis dimension; SD: sleep dimension.

## Data Availability

The data used to support the findings of this study are available from the corresponding author upon request.
